# The class-specific BCR tonic signal modulates lymphomagenesis in a *c-myc* deregulation transgenic model

**DOI:** 10.18632/oncotarget.2297

**Published:** 2014-07-31

**Authors:** Rada Amin, Abdelghafour Marfak, Céline Pangault, Christelle Oblet, Aurélie Chanut, Karin Tarte, Yves Denizot, Michel Cogné

**Affiliations:** ^1^ Centre National de la Recherche Scientifique, Limoges, France; ^2^ Université de Limoges, Limoges, France; ^3^ INSERM UMR U917, Rennes, France

**Keywords:** BCR, c-myc, lymphoma, IgA, B cell

## Abstract

Deregulation of *c-myc* by translocation onto immunoglobulin (Ig) loci can promote B cell malignant proliferations with phenotypes as diverse as acute lymphoid leukemia, Burkitt lymphoma, diffuse large B cell lymphoma, myeloma… The B cell receptor (BCR) normally providing tonic signals for cell survival and mitogenic responses to antigens, can also contribute to lymphomagenesis upon sustained ligand binding or activating mutations. BCR signaling varies among cell compartments and BCR classes. For unknown reasons, some malignancies associate with expression of either IgM or class-switched Ig. We explored whether an IgA BCR, with strong tonic signaling, would affect lymphomagenesis in *c-myc* IgH 3′RR transgenic mice prone to lymphoproliferations. Breeding *c-myc* transgenics in a background where IgM expression was replaced with IgA delayed lymphomagenesis. By comparison to single *c-myc* transgenics, lymphomas from double mutant animals were more differentiated and less aggressive, with an altered transcriptional program. Larger tumor cells more often expressed CD43 and CD138, which culminated in a plasma cell phenotype in 10% of cases. BCR class-specific signals thus appear to modulate lymphomagenesis and may partly explain the observed association of specific Ig classes with human B cell malignancies of differential phenotype, progression and prognosis.

## INTRODUCTION

In normal cells, *c-myc* oncogene expression is restricted to the early G_1_ phase of the cell cycle with a role in proliferation, differentiation, metabolism and apoptosis [1]. Deregulation of the *c-myc* gene is a constant feature of human Burkitt lymphoma (BL), with translocations linking *c-myc* to any of the immunoglobulin heavy or light chain (IgH, Igκ or Igλ) locus and with a phenotype that may vary from immature RAG expressing B cell lymphoma or leukemia to mature B cell lymphoma [2]. Translocation onto the IgH locus is also frequent in human myeloma and mouse plasmacytoma cases, suggesting that *c-myc* may participate to cell transformation at all stages of B cell differentiation. The frequency at which *myc*-driven malignancies occur clearly depends upon the cell microenvironment and the local production of cytokines. For example, inflammation-induced mouse plasmacytomas only develop in mice proficient for IL6 [3] and necessitates that these mice are kept in a conventional rather than specific pathogen free animal facility [4].

Beside oncogenes and the role of signals from the microenvironment, the phenotype of B cell malignancies also often involves signaling through the adaptive or innate immune receptors. The B cell receptor (BCR) is mandatory for survival of normal B cells by providing a ligand-independent tonic signal. It is also the main receptor controlling B cell activation during immune responses [5]. Expression of Ig of the various classes additionally has a clear influence on normal B cell fate [6-9].

In some cases of human lymphomas involving Epstein-Barr virus transformation, BCR-dependent activation or survival signals may be replaced by surrogate signals provided by the viral protein LMP2A [10]. Alternatively, in diffuse large B cell lymphomas (DLBCL), the activated B cell (ABC) phenotype features NF-κB activation due to somatic mutations at various levels of the BCR or TLR signaling cascades (Igα, Igβ, CARD11, Myd88…) and results in chronic BCR signaling in the absence of any BCR ligand [11, 12]. In other malignancies, ligand-dependent BCR signaling likely occurs. In follicular lymphoma, BCR molecules carrying high-mannose N-glycans may contribute to lymphomagenesis through their binding of C-type lectins in the tumor microenvironment [13]. In chronic lymphocytic leukemia (CLL), cell-autonomous signaling constitutively results from expression of self-binding BCRs [14]. Altogether, BCR signaling appears as strongly contributing to B cell transformation. As for untransformed B cells, the downstream effects of ligand-dependent or independent signals might be modulated according to the BCR class and to the stage at which oncogenic mutations immortalize a given cell. Interestingly, some B cell tumors overwhelmingly express IgM (as in CLL, follicular lymphoma, BL, lymphoplasmacytic lymphoma, ABC-type DLBCL, while others overwhelmingly express class-switched Ig (as in myeloma and GC-type DLBCL) [15]. It is thus questionable whether, similarly to their untransformed counterparts, malignant B cells could be drawn towards different phenotypes dependent about the Ig class produced.

We designed experiments in order to compare experimental B cell tumors induced by a similar oncogenic event, but in the context of either class-switched or IgM expression. We recently described a model of transgenic mice developing BL-like lymphomas with a mature B cell phenotype upon expression of a *c-myc* transgene. This transgene includes the four transcriptional enhancers (hs3a, hs1,2, hs3b and hs4) from the IgH locus 3′ regulatory region (3′RR), and is thus expressed at all stages of B cell differentiation, but with a higher activity at those stages undergoing terminal B cell differentiation and class switch recombination [16-21]. Transgenic animal carrying this *c-myc*3′RR transgene have been shown to develop BL in 75% of cases and diffuse anaplastic lymphomas in the remaining 25% [22]. Proliferations occurred with a still higher (100%) penetrance and were more heterogeneous in animal carrying both the *c-myc*3′RR transgene and p53 haploinsufficiency [23, 24]. Tumors from *c-myc*-3′RR mice were CD43 negative and had a mature B cell phenotype (B220^+^/IgM^+^/IgD^+^). In the present study, we introduced the *c-myc*3′RR transgene into a background carrying a homozygous mutation of the IgH locus (α1KI mice) [6-8]. This α1KI mutation corresponds to replacement of the Sμ region with a knock-in Cα IgH gene and replaces IgM/IgD expression by the premature expression of IgA. This forced expression of an IgA “class-switched” BCR has been shown to mediate constitutive pre-activation of B cells, even in the absence of any BCR ligation [6-8]. The present study was conducted by comparing single mutant (*c-myc* transgenics) with double mutant *c-myc*/α1KI animals. We thus monitored the occurrence of B cell malignancies upon the influence of both *c-myc* oncogene deregulation and class-switched-type constitutive BCR signaling and analyzed the phenotype of the observed malignancies.

## RESULTS

### Generation of double mutant α1KI *c-myc3*′RR mice

A colony of homozygous α1KI animals was generated, all bearing a single copy of the integrated *c-myc* cassette [22]. Single mutant α1KI homozygous mice were studied in parallel, as well as single *c-myc* transgenic animals.

### B cell development and Ig secretion in young transgenic mice

We analyzed B cells in 6 weeks-old transgenic mice, before any manifestation of disease. Spleen and lymph nodes (LNs) from α1KI mice and α1KI *c-myc*3′RR mice were similar (not shown). Numbers of B cells did not differ between homozygous α1KI mice and the double transgenic α1KI *c-myc*3′RR, neither in spleen (6.7 ± 1.7% N=5 for α1KI *versus* 8.9 ± 2.1% N=5, *i.e.* 1.4 ± 0.4 10^6^ cells *vs* 1.3 ± 0.19 10^6^ cells), nor in LNs (5.1 ± 0.8%, n=3 *vs* 5.3 ± 1.2%, n=3, *i.e.* 0.62 ± 0.09 10^6^ cells *vs* 0.44 ± 0.14 10^6^ cells) (supplemental Fig 1A). By contrast, *in vitro* B cell proliferative responses to anti-CD40 plus IL4 were evaluated and proved higher in α1KI *c-myc* transgenic mice than in α1KI single mutant animals (supplemental Fig 1B).

### Delayed lymphomas in α1KI *c-myc3*′RR mice vs *c-myc3*′RR single transgenics

**Figure 1:**
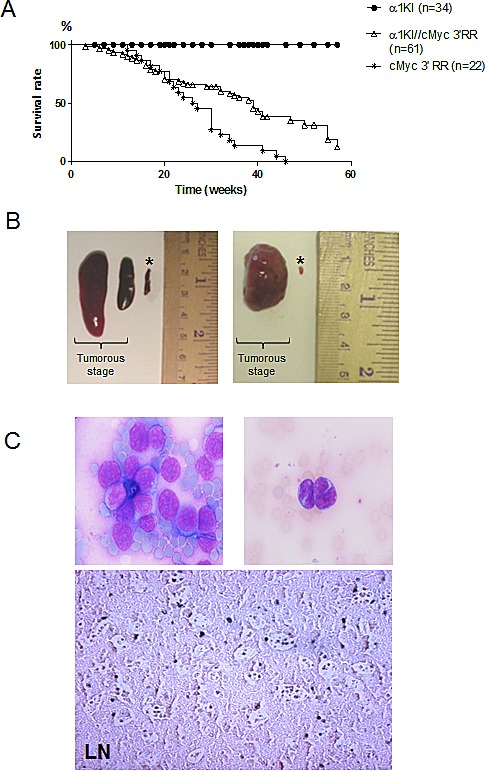
Generation of α1KI *c-myc3*′ RR transgenic mice (A) Survival curves of *c-myc*3′RR and α1KI *c-myc*3′RR mice. Twenty two *c-myc*3′RR mice (*), 61 α1KI *c-myc*3′RR transgenic mice (Δ) and 34 α1KI (•) mice were followed over a period of 60 weeks. (B) Enlarged organs from α1KI *c-myc*3′RR mice at the tumor development stage compared with organs from a control (*) α1KI *c-myc*3′RR at the pre-tumorous stage (Left: 2 representative tumorous spleens; right: 1 representative tumorous LN) (C) Histological features of tumors in double transgenic mice: (Up) Typical circulating tumor cells with a basophilic cytoplasm (original magnifications, x20). (Down) Sections of formalin-fixed, paraffin-embedded LNs were stained with hematoxylin-eosin.

Lymphoma development and mean lifespan were quoted for 61 double mutant α1KI *c-myc*3′RR transgenic mice, 34 single mutant α1KI homozygous mice and 22 single mutant *c-myc*3′RR animals. The overall tumor incidence in double mutant animals was 48% before 34 weeks of age. As expected by comparison to α1KI mice not susceptible to lymphoma, α1KI *c-myc*3′RR mice showed significantly increased mortality (Gehan Breslow Wilcoxon test, p <0.0001) that resulted from *c-myc-*driven tumor development, with the mean age of death at about 39 weeks (Fig 1A). This was significantly below the mortality observed in *c-myc*3′RR single transgenic mice (Gehan Breslow Wilcoxon test, p <0.001), among which 80% had developed tumors before 34 weeks of age (with mean age of death at 27 weeks).

At necropsy, the α1KI *c-myc*3′RR had enlarged spleens, LNs and eventually liver (Fig 1B). All mice with tumors showed leukemic peripheral blood involvement with large circulating nucleolated lymphoma cells (Fig 1C, up). Histological analysis of tumors showed a “starry-sky” pattern reminiscent of human BL (Fig 1C, down).

### Phenotype of B cell lymphomas in α1KI *c-myc3*′RR mice

FACS analyses of 29 tumors from α1KI *c-myc*3′RR transgenics revealed two kinds of tumors, either lymphocytic (type I) or plasmablastic (type II).

**Figure 2:**
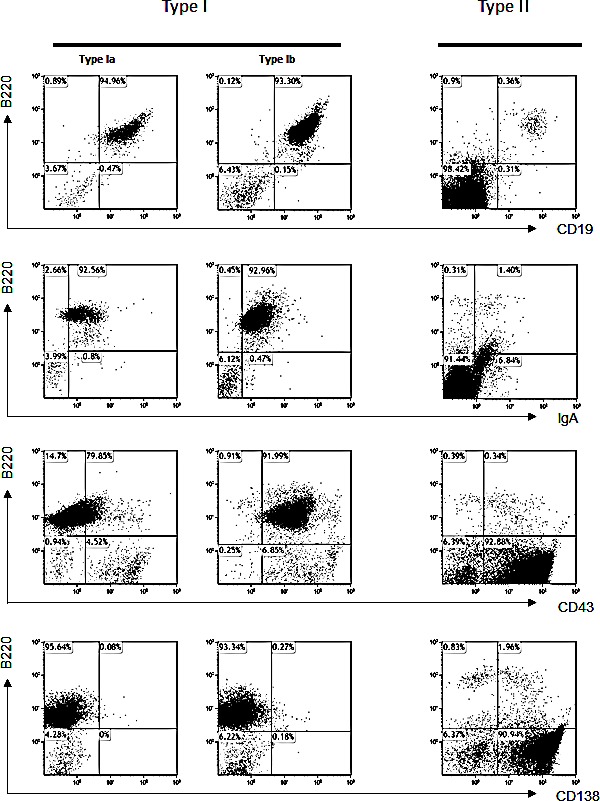
Analysis of lymphomas in α1KI *c-myc3*′ RR mice Tumors from 29 mice were stained with different cell surface markers and felt into 3 tumor types, for each of which one representative case is shown: Ia: CD19^+^/B220^+^/human IgA^+^/CD43^−^/CD138^−^; Ib: CD19^+^/human IgA^+^/B220^+^/CD43^+^/CD138^−^ and II: B220^−^/CD138^+^.

Lymphocytic (type I) tumors were the most frequent. They histologically resembled those tumors seen in *c-myc* single transgenics but could be further split in two subgroups according to CD43 expression: type Ia, IgA^+^/CD19^+^/B220^+^/CD43^−^ (Fig 2, left, 9 cases) and type Ib, IgA^+^/CD19^+^/B220^+^/CD43^+^ (Fig 2, middle, 17 cases).

Plasmablastic (type II) tumors occurred less frequently (3 cases, *i.e.* roughly 10%). They strongly differed from those tumors reported in *c-myc* single transgenics and corresponded to malignant plasmablasts (with an IgA^−^/CD19^−^/B220^−^/CD43^+^/CD138^+^ phenotype) (Fig 2, right, 3 cases).

**Figure 3:**
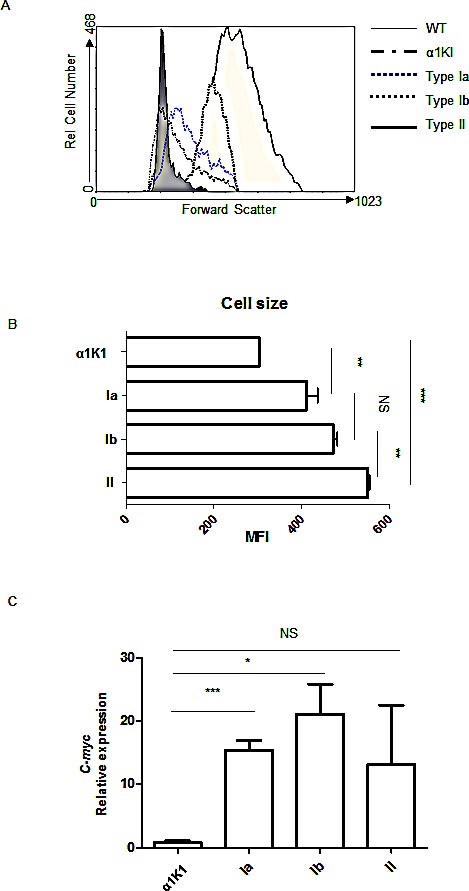
Tumor cell variations according to size and c-myc expression: (A) Total cells from α1KI c-myc3′RR tumors or normal B cells from α1KI and *wt* control mice were stained with PC5-conjugated anti-B220, PE-conjugated anti-CD19 (Type Ia and Ib) or PE-conjugated anti-CD138^+^ (type II) and analyzed by cell cytometry Shown is the forward scatter (FSC, directly evaluating cell size) of cells gated as [B220^+^/CD19^+^] for *wt*, α1KI, type Ia and Ib tumor B cells, or gated as [CD138^+^] for type II tumors. (B) Histograms showed the X-median of FSC gated as [B220^+^/CD19^+^] cells for control and type Ia and Ib tumors, and gated on [CD138^+^] cells for type II tumors (n=4). (C) Quantitative PCR analysis of *c-myc* expression for tumors of type Ia (n=5), type Ib (n=5) and type II (n=2) compared to control splenocytes from α1KI (n=3). The relative expression is shown as bar graph. (NS, not significant; *, p <0.05; ***, p <0.001, unpaired t-test)

By comparison to polyclonal B cells from pre-malignant animals, all lymphoma cases featured large B cells, with mean cell size increasing from type Ia to type Ib and to type II tumors (Fig 3A and B).

Lymphomas that developed in α1KI *c-myc*3′RR mice were checked for monoclonality by southern blot analysis of IgH gene configuration on tumor DNA samples taken at two different organ locations in each mouse. DNA from spleens of healthy α1KI *c*ontrols showed the germline IgH band, whereas all tumors displayed rearranged bands with a monoclonal pattern (supplemental Fig 2).

The 29 α1KI*c-myc*3′RR tumors analyzed all overexpressed *c-myc* at the mRNA level (Fig 3C). We checked several α1KI *c-myc*3′RR tumors of either tumor subtype for potential mutations within *c-myc* and found it un-mutated in all cases analyzed.

**Table 1:** List of genes up-regulated and down-regulated in tumors of α1KI c-myc3′RR vs c-myc3′ RR mice, according to their biological function

Category of genes	Up	Down
Ribosome biogenesis and protein synthesis	24	9
Transcription factors and DNA-binding proteins	16	5
Highly expressed in immune system	9	20
Intracellular signal transduction modulators and effectors	10	28
metabolic enzyme-related proteins	2	16
Lipid metabolism	6	7
Cell cycle	2	4
Apoptose	4	0
Cell-adhesion-related proteins	2	6
Blood coagulation proteins	0	4
Mitochondrial-related protein	5	2
Others	17	15
TOTAL	97	116

### Transcriptome analysis in α1KI *c-myc3*′RR lymphomas

The rare type II plasmablastic tumors arising in double transgenics obviously differed phenotypically from those seen in single *c-myc* transgenics and were not further explored at the mRNA level. Regarding type I lymphomas which were predominant and histologically reminiscent of those from *c-myc* single transgenic mice, we wondered whether subtle changes would be found by more in-depth molecular analyses. To thoroughly compare double mutant *vs* single *c-myc* transgenic mice tumors, we thus analyzed gene expression profiles of 8 IgA-expressing type I lymphomas (including 4 type Ia and 4 type Ib tumors) from double mutant mice compared to 8 IgM-expressing *c-myc*3′RR lymphomas (including 4 “BL-like” and 4 anaplastic lymphomas). Analysis was performed to identify differentially expressed genes, either significantly up-regulated or down-regulated in tumors from both groups of mice (Table 1 and supplemental Table 1). As previously described, lymphomas from *c-myc* single transgenics (whether BL-like or anaplastic) had roughly homogeneous profiles (Supplemental Fig 3) [22]. By contrast, the 8 α1KI tumors expressing an IgA BCR featured a clearly different gene expression profile, with 97 genes found up-regulated while 116 were down-regulated (both in type Ia and Ib tumors, whose profiles were roughly similar despite the differences regarding cell size and CD43 surface expression) (Table 1, Supplemental Fig. 3). Genes were classified according to their biological function (Table 1) and we used Ingenuity Pathway Analysis (IPA; Ingenuity Systems) to identify pathways that were enriched in the 2 lists reported in supplemental Table 2. The largest category of up-regulated genes involved ribosome biogenesis and protein synthesis (Table 1). These processes notably modulate cell growth and proliferation, and are frequently deregulated in cancer by influencing expression of oncogenes and tumor suppressors [28]. By itself, *c-myc* overexpression is known to stimulate ribosome assembly and result in increased cell size and protein synthesis [29].

Three genes negatively controlling cell proliferation were also strongly up-regulated in IgA-expressing tumors and may participate in delaying lymphomagenesis: the *GADD34/ Ppp1r15a* gene (up-regulated 12 fold) controls growth arrest during the unfolded protein response (UPR) [30]; *Gas5* (overexpressed 12 fold) is transcribed into a noncoding RNA that induces growth arrest [31], while *Btg1* (up-regulated 5 fold) is another inhibitor of cell proliferation [32]. By contrast, 4 down-regulated genes are tumor suppressors (*Lxn, Rassf4, Dusp16, Ifitm2*) [33-37].

Variations of another 3 genes could be connected with B cell activation: CD69 was overexpressed 4 fold in IgA tumors (similar to its overexpression polyclonal α1KI B cells) [6, 38]); *Trim12* (a repressor of lymphocyte activation) [39] was down-regulated 27 fold in IgA+ tumors, so as the gene for MARCH1, an E3 ubiquitin protein ligase targeting some membrane receptors for degradation and down-regulated by B cell activation [40].

Some other changes were related to membrane receptor expression or membrane trafficking, with notably a 3 fold increase in expression of the EHD2 ATP-ase, which stabilizes caveolae at the plasma membrane [41], and down-regulation of 15 other genes involved in related functions (*Slc7a7, Slc39a4, Ggh, Sirpa, Rap1gap, H2afb3-ps, Slc37a2, Ctsd, Arhgap9, Gng12, Eml2, Ctsh Gpr18*). Expression of Fcγr4 and of the γ chain of FcγR1 was also down-regulated.

Some changes were related to signal transduction and may notably increase tyrosine kinase and *wnt* signals in IgA-expressing tumors, with 5 fold increased *Lgr5* (an activator of *wnt* signaling) [42] and a 3 fold down-regulation of two *wnt* inhibitors, *Trim59* (a repressor of *c-myc*-dependent *wnt* induction) [43] and the CD9 tetraspanin which inhibits *wnt*/β-catenin signalization) [44]. Interestingly, two tyrosine kinase inhibitors are down regulated by 12 fold in IgA tumors: *Lst1* a membrane adaptor recruiting phosphatases SHP1 and SHP2, and the *DAP12* gene encoding tyrosine kinase binding protein TyroBP [45, 46]. *NFATc2*, a transcription factor involved in B cell anergy and whose defect has been correlated with B cell hyper-responsiveness is down-regulated 5 fold [47]. We also noticed a 4-fold increased expression of *Dusp6*, an inhibitory MAP kinase phosphatase, down-regulation (10-fold) of *Nafm1*, an ITAM receptor that modulates BCR signaling [48] and of *Hck*, a src family kinase expressed in B cell progenitors [49].

Another major gene category included additional transcription factors, DNA-binding proteins and histones, with strong up-regulation of the *Fos-B* transcription factor gene (FBJ osteosarcoma oncogene B) (P= 9.95E-05). Fos family proteins form heterodimers with jun family proteins and constitute AP-1 transcriptions factors. These factors regulate the expression of multiple genes modulating cell proliferation, activation, differentiation and eventually cell death [50-53].

Interestingly, Ingenuity Pathway Analysis of down-regulated genes pointed out that significantly enriched biologic functions were linked to cellular movement, immune cell trafficking and hematological system development and function (supplemental Table 2).

Finally, some variations might be correlated with the control of the tumor niche and of anti-tumor immunity, such as *ebi3*, a subunit of IL27 normally over-expressed in centrocytes (and down 16 fold in IgA tumors) [54], CCL9 (down 10 fold in IgA tumors), a chemokine known to attract CCR1-expressing stromal cells and also expressed in macrophages and myeloid cells [55, 56], the *nab2* transcription factor (known to stimulate TRAIL expression) down-regulated 6 times [57], the 10-fold overexpressed *pvrl2* (an inhibitor of NK cytotoxicity) [58], the 3-fold overexpressed cortistatin (an immunomodulatory anti-inflammatory peptide produced by immune cells) [59], and the 2.8-fold overexpressed CCL25, a chemokine normally attracting lymphocytes to the small bowel [60].

## DISCUSSION

Expression of the BCR is a major regulator of B cell fate, with signaling thresholds that vary between B1, follicular or marginal zone B cells. The tonic signal provided by BCR expression without any need for an extra-cellular ligand is mandatory for B cell survival and has also been reported as increased by class switching to the IgG or IgA class [6, 8, 61].

All along B lymphocyte development, cell survival is dependent from the production of Ig as membrane-anchored receptors (mIg). That even plasma cell survival might somehow benefit from soluble Ig production is still debated [62-64]. While of major importance in physiology, the role played by BCR signaling and Ig production with regard to the maintenance or growth of malignant B cells is still incompletely appreciated. Except for very immature proliferations, most cases of B cell malignancies feature expression of Ig, either in the form of a membrane-anchored pre-BCR / BCR for lymphomas or as secreted Ig for plasma cell proliferations. In some types of malignancies, the biased use of canonical V (D) J rearrangements suggests the role of chronic antigen/BCR stimulation [65]. Some other lymphoma cases involve deregulated signaling by mutated components of the BCR cascade [11]. The class of the BCR or of the secreted monoclonal Ig often correlates with the phenotype of human B cell malignancies, IgM expression being associated with less differentiated tumors [15, 66]. This raises the question of whether specific BCR properties might influence the tumor phenotype and supply different cell growth and/or survival signals. Beside the role of Ig-mediated tonic signals in cell survival, the ability of B cells to be activated and to differentiate relies on transient signals provided during BCR interaction with antigens. It is generally assumed that in immature B lymphocytes expressing IgM, early Ag contact leads to cell death or anergy while it promotes activation and differentiation of mature mIgM/mIgD-expressing B cells [67]. Also in class-switched mIgG^+^, mIgA^+^ or mIgE^+^ B cells and memory cells, BCR activation by antigen usually induces proliferation and differentiation into plasma cells. While all membrane Ig associate with the Ig-α / Ig-β complex to constitute the BCR, several studies have suggested that cells with a class-switched BCR hereby undergo some phenotypic changes. In particular, signaling from an IgG or IgA class BCR seems to induce more efficient B cell activation and short-term memory, while long-term accumulation as memory B cells might be a preferential feature of IgM expressing cells [8, 9, 61].

Among lymphomas, BL is characterized by the translocation of *c-myc* onto one of the Ig loci and the resulting *c-myc* deregulation then stands as the initial and driver hit [2]. It was shown in mice that the IgH locus 3′RR is instrumental for oncogenicity of c-myc translocations [68], which can also be artificially mimicked by associating a *c-myc* transgene with the IgH 3′RR [22]. The latter model induced two kinds of B cell neoplasias with different kinetics. Prevalent tumors overexpressed *c-myc* and arose at the mean age of 12 weeks with features resembling human BL: high proliferation, high level of apoptosis and a typical “starry sky” histological morphology, involving medium-sized mature B cells (B220^+^CD19^+^IgM^+^IgD^+^) with a basophilic cytoplasm. A second type of neoplasms arose at the mean age of 20 weeks, mostly involved liver and spleen, presented medium to large B220^neg^, CD138^neg^ cells and were classified as anaplastic lymphomas [22, 23, 69].

In the present study, we backcrossed the *c-myc*3′RR transgene in the α1KI genetic background expressing a modified BCR of the IgA class. Such an IgA BCR was previously shown to yield high constitutive signaling in the absence of any BCR ligation. The present study shows that in double α1KI *c-myc*3′RR transgenic animals, the frequency of *c-myc-*driven tumors is decreased, their progression is lowered and the incurring lymphomas appear as more differentiated or even committed (in 10% of cases) into terminal BCR^−^, CD43^+^, CD138^high^ plasma cell differentiation.

The slowed kinetics in the onset and progression of tumors is in agreement with the involvement of more differentiated cells. While all the tumors from the single transgenic mice were negative for both CD43 and CD138, most of double transgenic tumors have an activated phenotype with large CD43^+^ cells.

The *c-myc* oncogene has been described as a global regulator of various cell functions, and its overexpression usually results in tumorigenesis through a global deregulation of processes including increased cell proliferation, increased apoptosis, increased cell size, and marked changes in the total rate of protein synthesis [29, 70, 71]. In that regard, *c-myc* increases transcription of ribosomal RNA genes, recruitment of rRNA processing factors, ribosomal subunit export and recruitment of translation initiation factors [72]. In addition, it was shown that the *c-myc*3′RR transgene yielded more diverse tumor phenotypes (including mantle cell lymphoma, BL and plasma cell lymphoma) upon association to other deregulated oncogenic processes in *cdk4* [24, 73] or *p53* [23] mutant mice.

In the present study, the IgA class BCR modifies c-myc–driven tumorigenesis by modulating genes involved in distinct biology function, then stimulating cell activation and differentiation while inhibiting cell growth. and further increasing the ribosome biogenesis and protein synthesis already targeted by c-myc. Interestingly, 3 of the genes up-regulated in IgA+ mouse lymphomas have already been underlined in human lymphomas as among the 6 genes suggested as sufficient to distinguish GC-type from ABC-type DLBCLs [74]. These genes are GADD34 (up-regulated 12-fold), Myosin1 (up-regulated 5.9 fold) and fibronectin-1 (up-regulated 3.9 fold). Such genes up-regulated both in GC-type vs ABC-type DLBCL and in IgA+ vs IgM+ mouse lymphomas, are reminiscent of the observation that GC-type DLBCLs mostly express class-switched BCRs, while ABC-type DLBCLs mostly express IgM [14].

Altogether, IgA tumors in α1KI *c-myc*3′RR double mutant animals have a modified phenotype by comparison to IgM tumors from single *c-myc* transgenic mice, showing that a class-switched BCR signal resulted in cell transformation with a more differentiated or activated lymphoid phenotype or even increased occurrence of plasma cell phenotypes, then reminiscent of human plasma cell proliferations that overwhelmingly produce class-switched Ig. This could also be pertinent to the observations made in patients, where IgM expressing malignancies are usually more aggressive than related class-switched proliferations, as noticed for the rare IgM myelomas, with lymphoplasmacytic rather than plasmacytic proliferations or as noticed for DLBCL, where the worse prognosis associates with the IgM-expressing ABC-type.

## MATERIALS AND METHODS

### Mice

All mice were in a C57Bl6 background previously shown to allow a nearly constant occurrence of *c-myc*-driven lymphomas within the first 9 months of life [22]. The *c-myc* transgene includes the murine *c-myc* as a 7-kb *Sma*I*-Kpn*I genomic fragment including P1 and P2 promoters, followed with the four IgH 3′RR enhancers: hs1,2 as a 0.6-kb *Stu*I-*Eco*RV fragment flanked on both sides with hs3a and hs3b as two 2.1-kb *Eco*RI-*Hind*III fragments cloned with symmetric orientations, and hs4 as a 1.38-kb *Pst*I-*Hind*III fragment [25]. This *c-myc* expression cassette is flanked by β-globin insulators. The α1KI mutation corresponds to insertion of a 6 kb IgH Cα1 human genomic fragment (from upstream of CH1α to a BamHI site downstream of the membrane-form transcript polyadenylation signal) in replacement of the mouse IgH Sμ region [6]. The project was approved by our local ethics committee review board. Mice were followed daily for clinical examination and macroscopic tumor development. Animal exhibiting important tumors or presenting severe signs of anemia or illness were sacrificed.

### Southern blot analysis

Genomic DNA was extracted from mice tails. Ten μg of DNA were digested with *Eco*RI, loaded on 0.7% agarose gels, transferred on nylon sheets (Amersham, Buckinghamshire, UK), hybridized with a ^32^P-labeled hs4 probe (a 1.3 kb *Pst*I fragment) and autoradiographed to ensure the presence of the *c-myc*3′RR transgene as previously reported [22].

Clonality of tumors was also assayed by southern blots of EcoRI digested tumor DNAs, with an IgH probe located around the JH4 segment (a 0.5kb SacI-NaeI fragment).

### *In vitro* stimulation assays

Splenic B cells from α1KI *c-myc*3′RR and α1KI homozygous control mice were stimulated for 3 days with 20 μg/mL of LPS from *Salmonella typhimurium* (Sigma, St-Louis, MO) or with 5 μg/mL anti-CD40 (R&D systems, Lille, France) plus 40 ng/mL murine IL4 (PeproTech) in RPMI supplemented with 10% heat-inactivated fetal calf serum. The number of viable proliferating cells was assessed using MTS solution [3-(4,5-dimethylthiazol-2-yl)-5-(3-carboxymethoxyphenyl)-2-(4-sulfophenyl)-2H-tetrazolium], and a colorimetric assay (Promega) following the manufacturer's recommendations.

### mRNA expression

Total RNA was extracted from fresh tissues and cell suspensions using tri-reagent (Ambion Austin, TX). RNA was reverse-transcribed into cDNA by addition of reverse transcriptase to 2 μg total RNA in a final volume of 20 μl. Real-time PCR was performed in duplicate by using TaqMan assay reagents and analyzed on an ABI Prism 7000 system (Applied Biosystems Foster City, CA). Transcripts analyzed were *c-myc* (Mm00487803-m1) and 18S RNA (Hs99999901-s1) used for gene expression level normalization (Applied Biosystems).

### Microarray preparations

RNA was extracted from hyperplasic lymph nodes (LNs). Experiments were done in quadruplicate and 0.2μg of RNA was used for each microarray analysis. cDNA labeling and dual color microarray hybridization were done in the Nice Sophia-Antipolis Microarray Facility (Nice Sophia Antipolis, France).

### Gene Data Expression Analyses

*Cluster analysis.* GenBank accession number - GSE 32229. Log 2 signals were extracted after interslide quantile normalization and performed with R (Bioconductor). Probe sets showing minimal variation across all samples were filtered out, only retaining those 3217 with a coefficient of variation over 0.1, and using them to cluster the samples through the hierarchical agglomerative algorithm. Average linkage and Euclidian distance were used as linkage and similarity methods, respectively. Supervised classification was carried out using Cluster and TreeView software.

*Detection of differentially expressed genes.* Statistical analyses used the R and Bioconductor software. To identify over- or under-expressed genes, we used the empirical Bayesian method for differential expression calculation [26] implemented in the Linear Models for Microarray Analysis (LIMMA) package. For each pairwise comparison, p-values were adjusted after multiple testing corrections using the Benjamini and Hochberg method [27]. Probes with a corrected *p* value below 0.05 were considered as differentially expressed.

Futhermore, gene lists of enriched pathways were generated using Ingenuity Pathway Analysis.

### Flow cytometry analysis

Cell suspensions from spleen, liver and LNs were stained with various antibodies (Southern Biotechnologies or eBiosciences): SpectralRed (PC5)-conjugated anti-B220, phycoerythrin-labeled (PE) anti-CD19, anti-CD138, FITC-conjugated anti-human IgA and PE anti-mouse CD43. Cells were analyzed on a Coulter XL apparatus (Beckman Coulter, Fullerton, CA).

### Blood sampling

Blood samples were collected from transgenic and wild-type (*wt*) mice with heparinised needles. Blood smears allowed differential counting of blood cells after staining by the May-Grünwald-Giemsa method.

## SUPPLEMENTARY MATERIAL, TABLES AND FIGURES


