# Supplementary material for: The class-specific BCR tonic signal modulates lymphomagenesis in a c-myc deregulation transgenic model
Source: Oncotarget. 2014 Jul 31;5(19):8995–9006. doi: 10.18632/oncotarget.2297 (PMC4253413; doi:10.18632/oncotarget.2297)
Supplement: Supplementary file 1 [file oncotarget-05-8995-s001.pdf]

The class-specific BCR tonic signal modulates lymphomagenesis in a *c-myc* deregulation transgenic model

Supplementary Material

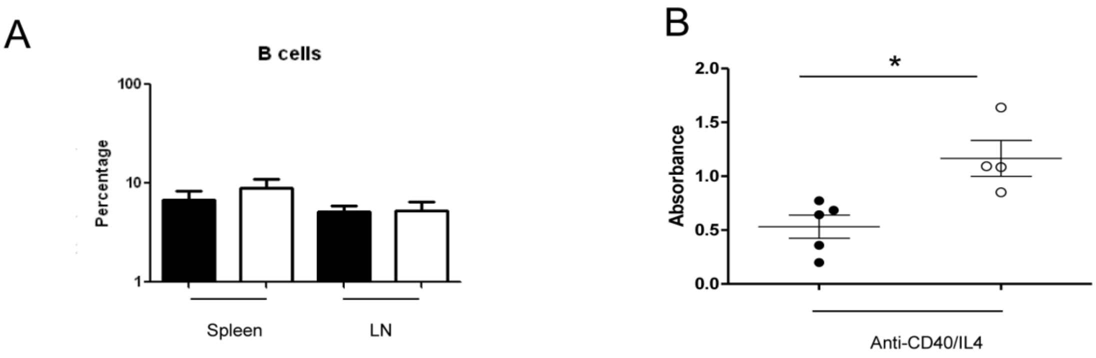

Supplemental Figure 1: Development and proliferation of B cell in α1KI *c-myc*3'RR

(A) Evaluation of B cells in spleen (n=5) and LNs (n=3) from 6 weeks old mice before any disease development. Percentage of $B220^+$ cells from α1KI *c-myc*3'RR (white bars) compared to α1KI (black bars). (NS, not significant; *, p <0.05) **(B)** Splenocytes from the same α1KI *c-myc*3'RR (white circles) and α1KI/*c*ontrol mice (black circles) were stimulated *in vitro* with LPS or with anti-CD40 plus IL4 for 4 days. Absorbance in the MTS assay evaluates proliferation. Results from 5 independent experiments are shown. (NS, not significant; *, p <0.05, unpaired t-test).

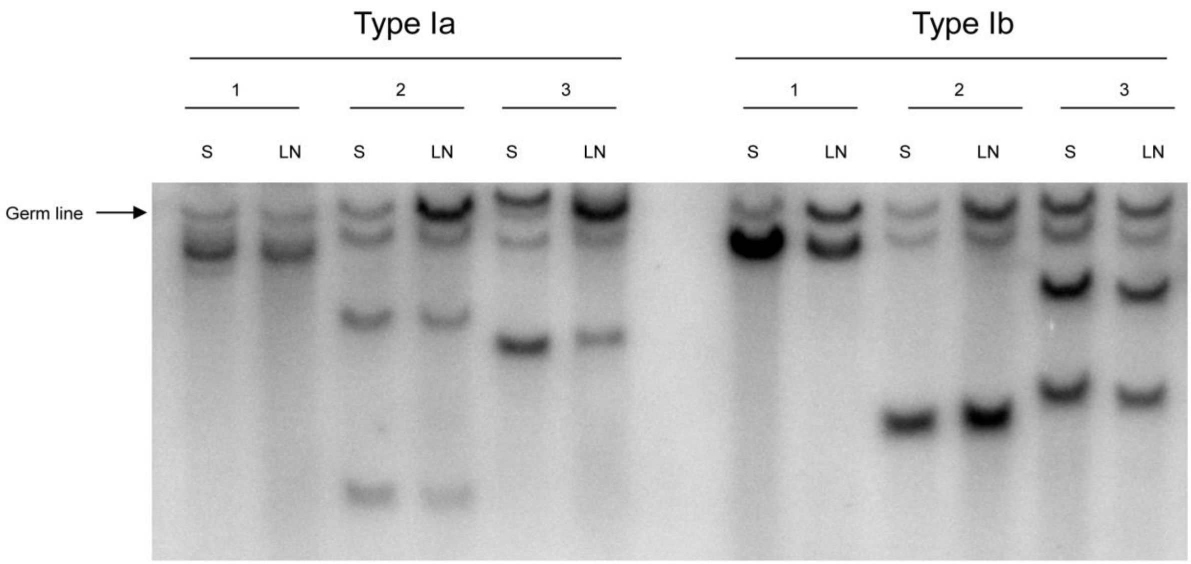

Supplemental Figure 2: Clonality of lymphomas in α1KI *c-myc*3'RR mice Southern blot analysis was used to check lymphoma clonality with a JH4 probe. Genomic DNA from hyperplasic spleens (S) and LNs were prepared and digested with EcoRI from different tumors (3 individual mice per group, each analyzed for spleen and LN).

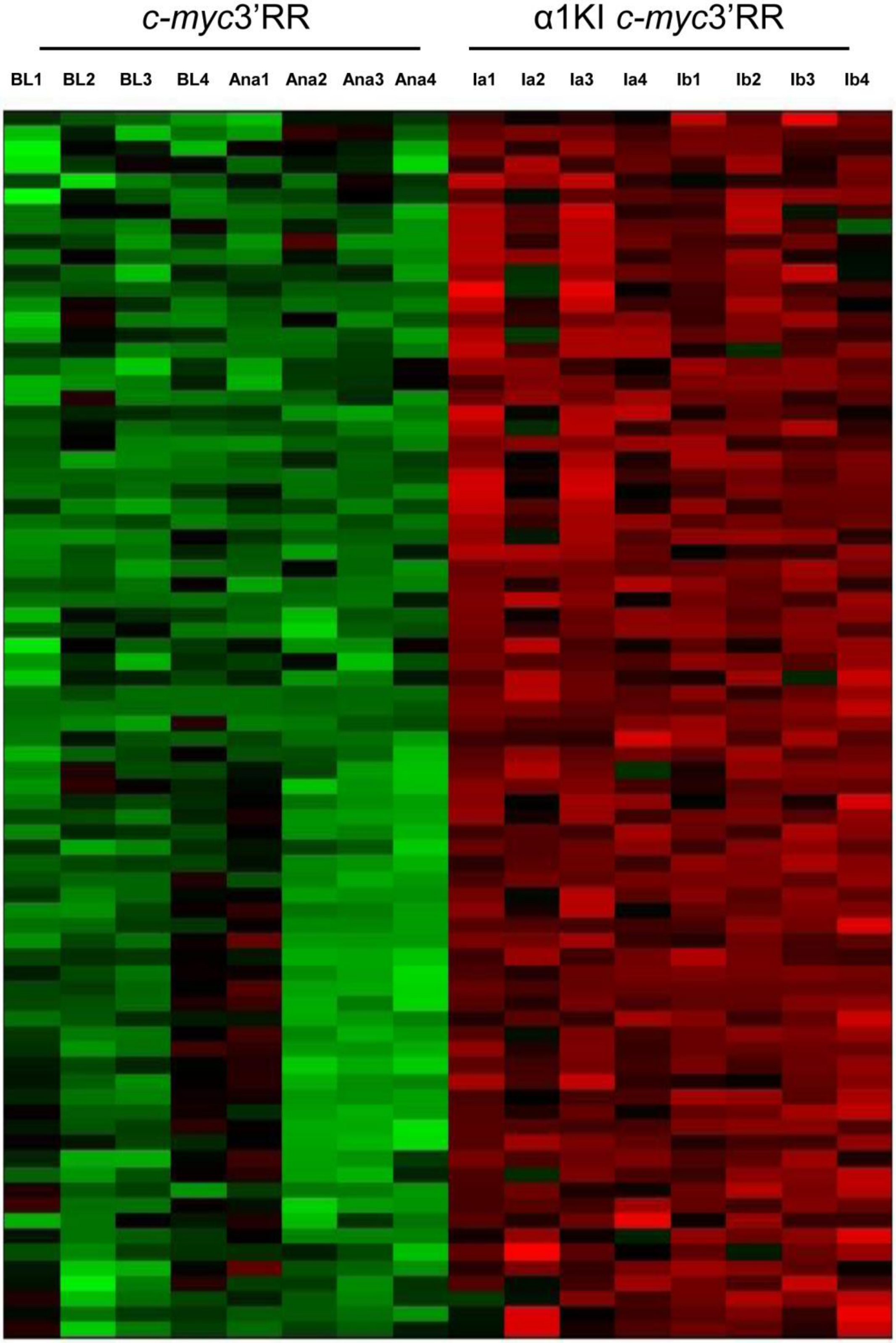
c-myc3'RR
α1KI c-myc3'RR
BL1 BL2 BL3 BL4 Ana1 Ana2 Ana3 Ana4
Ia1 Ia2 Ia3 Ia4 Ib1 Ib2 Ib3 Ib4

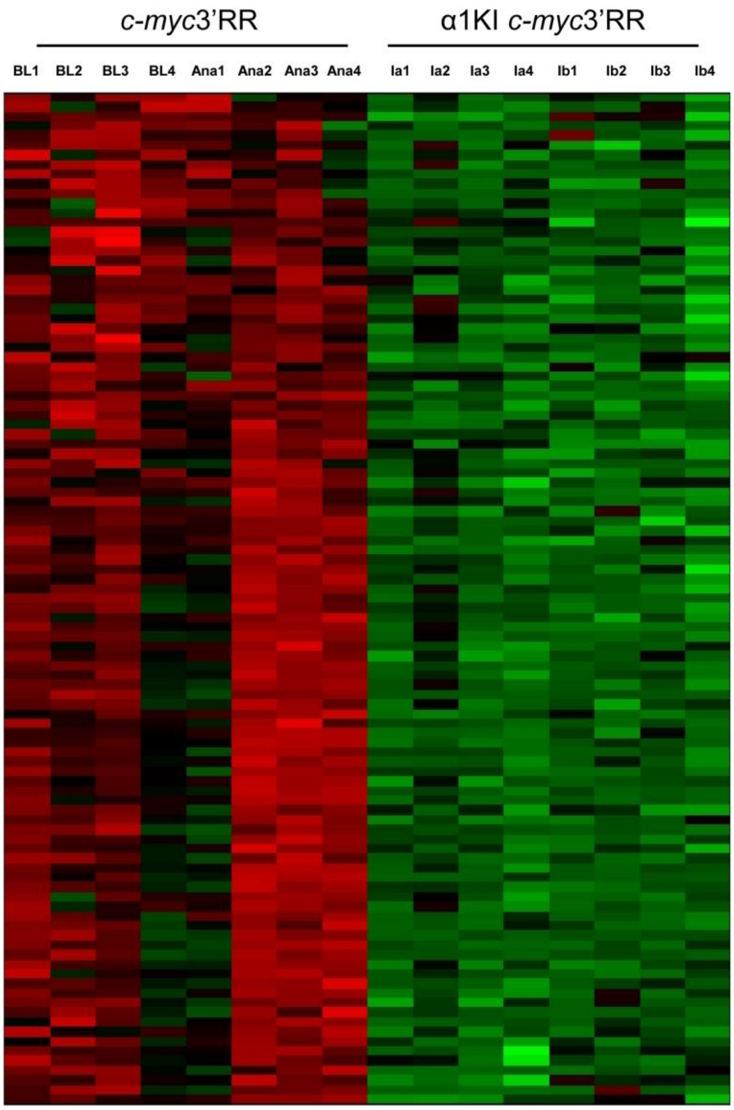

Supplemental Figure 3: RNA profiling of type Ia and Ib double transgenic tumors *vs* "Burkitt-like" (BL) and anaplastic (ANA) tumors from single transgenic *c-myc* mice: The clustered genes shows the most significantly genes up-regulated (A) and down-regulated (B) in type Ia (n=4) and type Ib (n=4) from α1KI *c-myc*3'RR tumors *vs* *c-myc*3'RR. Red represents up-regulation of gene expression, and green represents down regulation of gene expression.

Supplemental Table 1: RNA profiling of type Ia and Ib α1KI *c-myc*3'RR tumors compared to *c-myc*3'RR lymphomas. Data for the 213 most different genes were reported. The up-regulated (A) and down-regulated (B) genes in type Ia and Ib *vs* *c-myc*3'RR were represented in two different sub-tables. Columns include gene names, gene descriptions, absolute fold-change (FC), signal ratio between double transgenic and single transgenic tumors and P-values.

Supplemental Table 1 (A) : Up-regulated genes

Gene symbol	Gene name	Absolute (FC)	Log(FC)	adjust p-Value
Ribosome biogenesis and protein synthesis				
Tbc1d2	TBC1 domain family, member 2	5,84	2,55	5,68E-02
Tbc1d2	TBC1 domain family, member 2	4,77	2,25	5,89E-02
Gm14131	similar to chloride channel, nucleotide-sensitive, 1A	4,19	2,07	1,24E-02
Rpl21	ribosomal protein L21	3,97	1,99	1,02E-04
Rnu1b6	U1b6 small nuclear RNA , small nuclear RNA	3,83	1,94	2,88E-02
Ankrd34a	ankyrin repeat domain 34A	3,56	1,83	4,17E-05
Enpp4	ectonucleotide pyrophosphatase/phosphodiesterase 4	3,45	1,78	4,37E-02
ENSMUST00000117580	ubiquitin A-52 residue ribosomal protein fusion product 1 Gene	2,82	1,49	8,11E-02
ENSMUST00000119783	GATA zinc finger domain containing 1 Gene	2,74	1,45	2,60E-02
Gm14052	similar to heterogeneous nuclear ribonucleoprotein A3	2,69	1,43	3,41E-02
Rpl17	ref|Mus musculus ribosomal protein L17	2,52	1,33	5,41E-03
Gm4510	similar to ribosomal protein S19	2,47	1,31	2,69E-03
Gm9179	similar to ribosomal protein L32	2,44	1,29	6,07E-03
Gm8880	similar to ribosomal protein L21	2,34	1,22	7,02E-03
Gm2334	similar to Ubtf protein	2,32	1,22	9,32E-02
Gzmm	granzyme M (lymphocyte met-ase 1)	2,31	1,21	1,70E-02
LOC637851	similar to heterogeneous nuclear ribonucleoprotein A3	2,24	1,17	3,02E-02
LOC674157	hypothetical LOC674157	2,24	1,16	2,56E-02
4930565O14	tRNA-guanine transglycosylase {Homo sapiens}	2,20	1,14	6,24E-03
Dtymk	deoxythymidylate kinase , transcript variant 2	2,18	1,12	8,42E-02
Rrp8	ribosomal RNA processing 8, methyltransferase, homolog (yeast)	2,02	1,02	3,68E-02
Eif4a2	eukaryotic translation initiation factor 4A2 , transcript variant 1	2,05	1,04	4,81E-02
Eif2s1	eukaryotic translation initiation factor 2, subunit 1 alpha	5,31	2,41	1,16E-02
Rg9mtd2	RNA (guanine-9-) methyltransferase domain containing 2, full insert sequence	2,27	1,18	1,53E-02
Transcription factors and DNA-binding proteins				
Fosb	FBJ osteosarcoma oncogene B	52,30	5,71	9,95E-05
Hist1h2bg	histone cluster 1, H2bg	8,29	3,05	2,77E-02
H3f3b	H3 histone, family 3B	4,49	2,17	6,28E-02
Hist2h2bb	histone cluster 2, H2bb	4,01	2,00	8,17E-02
Zfp608	zinc finger protein 608	3,51	1,81	3,18E-02
Hist1h1e	histone cluster 1, H1e	2,79	1,48	7,01E-02
Hist1h3f	histone cluster 1, H3f	2,72	1,44	9,72E-02
Gm12260	similar to histone H3	2,28	1,19	2,25E-02
Kbtbd12	kelch repeat and BTB (POZ) domain containing 12 , transcript variant 1	2,27	1,18	3,21E-02
Satb2	special AT-rich sequence binding protein 2	2,28	1,19	3,65E-02
Tiparp	TCDD-inducible poly(ADP-ribose) polymerase	2,24	1,16	6,24E-03
Hist1h2aa	histone cluster 1, H2aa	2,18	1,13	2,40E-02
Atad2	ATPase family, AAA domain containing 2	2,18	1,12	7,77E-02
Hist1h3d	histone cluster 1, H3d	2,11	1,08	2,34E-02
Kbtbd11	kelch repeat and BTB (POZ) domain containing 11	2,56	1,36	7,48E-02
H3f3b	H3 histone, family 3B	2,15	1,10	2,45E-02
Highly expressed in immune system				
Gm8909	predicted gene 8909	11,46	3,52	2,25E-03
Klra16	killer cell lectin-like receptor, subfamily A, member 16	4,15	2,05	6,07E-03
Cd69	CD69 antigen	3,68	1,88	6,27E-02
Ccl25	chemokine (C-C motif) ligand 25 (Ccl25), transcript variant 1	2,80	1,48	1,24E-02
LOC235882	similar to Ifi203 protein	2,38	1,25	6,30E-02
F630048H11Rik	NOD-derived CD11c +ve dendritic cells cDNA, RIKEN full-length enriched library	2,26	1,18	8,93E-02
Klra22	killer cell lectin-like receptor subfamily A, member 22	2,08	1,06	9,86E-02
Gm9442	similar to prothymosin alpha	2,08	1,06	5,16E-02
Intracellular signal transduction modulators and effectors				
Dusp6	dual specificity phosphatase 6	3,96	1,98	5,65E-02
Ehd2	EH-domain containing 2	3,13	1,65	7,77E-02
Dnahc8	dynein, axonemal, heavy chain 8	2,94	1,56	6,74E-02
Dnajb9	DnaJ (Hsp40) homolog, subfamily B, member 9	2,47	1,31	8,31E-02
Ptk2	PTK2 protein tyrosine kinase 2 , transcript variant 2	2,37	1,24	6,22E-02
Tram2	translocating chain-associating membrane protein 2, transcript variant 2	2,20	1,14	7,77E-02
Tmod4	tropomodulin 4	2,10	1,07	8,89E-02
Gpd1l	glycerol-3-phosphate dehydrogenase 1-like	2,09	1,06	8,56E-02
Pkib	protein kinase inhibitor beta, cAMP dependent, testis specific , transcript variant 2	2,08	1,06	8,41E-02
Metabolic enzyme-related proteins				
B3galtl	beta 1,3-galactosyltransferase-like	2,47	1,31	7,16E-03
B4galt3	UDP-Gal:betaGlcNAc beta 1,4-galactosyltransferase, polypeptide 3	3,96	1,98	7,48E-03
Lipid metabolism				
Lgr5	leucine rich repeat containing G protein coupled receptor 5	4,83	2,27	7,40E-02
LOC100046908	similar to cyclic nucleotide gated channel beta 1	3,17	1,66	6,71E-02
ENSMUST00000101612	ATP-binding cassette, sub-family B, member 4 Gene	3,02	1,59	1,95E-02
Abcb4	ATP-binding cassette, sub-family B (MDR/TAP), member 4	2,66	1,41	6,98E-02
Hsd17b7	hydroxysteroid (17-beta) dehydrogenase 7	2,07	1,05	2,41E-02
Cell cycle				
Rbl1	retinoblastoma-like 1 (p107) (Rbl1), transcript variant 2	2,10	1,07	5,65E-02
Cort	cortistatin (Cort)	3,12	1,64	3,13E-02
Apitd1	apoptosis-inducing, TAF9-like domain 1	2,68	1,42	4,05E-02
Apoptose				
Ppp1r15a	protein phosphatase 1, regulatory (inhibitor) subunit 15A	12,64	3,66	5,64E-03
Gas5	growth arrest specific 5 (Gas5)	12,61	3,66	6,68E-03
Btg1	B-cell translocation gene 1, anti-proliferative	5,24	2,39	1,36E-02
Cell adhesion-related protein				
Pvrl2	poliovirus receptor-related 2 , transcript variant 2	4,35	2,12	7,18E-03
Pvrl2	poliovirus receptor-related 2 , transcript variant 1	9,45	3,24	4,81E-03
Mitochondrial -related protein				
Dars2	aspartyl-tRNA synthetase 2 (mitochondrial)	2,09	1,06	6,24E-03
Slc25a37	solute carrier family 25, member 37 , nuclear gene encoding mitochondrial protein	2,56	1,36	6,97E-02
Gm16399	similar to cytochrome c oxidase, subunit Vic	2,32	1,21	5,64E-03
Mcart1	mitochondrial carrier triple repeat 1 , nuclear gene encoding mitochondrial protein	2,28	1,19	6,24E-03
ND6	mitochondrially encoded NADH dehydrogenase 6 Gene	2,22	1,15	8,70E-02
Others (Table 1-suite)				
4931429I11Rik	RIKEN cDNA 4931429I11 gene	31,75	4,99	9,95E-05
Gm1983	similar to CG15780-PA (LOC100038956)	4,19	2,07	2,69E-03
ENSMUST00000118228	tribbles homolog 1 Gene	3,22	1,69	4,34E-02
C530028O21Rik	RIKEN cDNA C530028O21 gene	2,93	1,55	3,21E-02
LOC100048452	hypothetical protein LOC100048452	2,92	1,55	8,20E-02
Gm6323	predicted gene, EG622446 (EG622446)	2,92	1,54	4,81E-03
6230415J03Rik	11 days embryo head cDNA, RIKEN full-length enriched library	2,70	1,43	1,24E-02
E130304I02Rik	activated spleen cDNA, RIKEN full-length enriched library	2,67	1,42	9,96E-02
Gm12118	similar to Ac2-008 (LOC666269)	2,66	1,41	6,68E-03
EG638052	predicted gene, EG638052 (EG638052)	2,53	1,34	6,68E-03
Krtap8-2	keratin associated protein 8-2 (Krtap8-2)	2,53	1,34	5,41E-03
Gm9456	hypothetical LOC669389 (LOC669389)	2,46	1,30	3,46E-02
1110002E22Rik	similar to hCG2038359 (LOC100039528)	2,40	1,26	4,28E-02
Gm13154	predicted gene 13154 (Gm13154)	2,40	1,26	4,43E-02
Gm7721	predicted gene, EG665631	2,21	1,15	3,99E-02
D16Ertd472e	DNA segment, Chr 16, ERATO Doi 472, expressed	2,18	1,12	1,33E-02
Gm3219	predicted pseudogene 3219	2,12	1,08	6,22E-02
Gm2862	hypothetical protein LOC100045710	2,07	1,05	6,49E-02
Gm5196	predicted gene, EG382769	2,06	1,04	6,56E-02
C030048B08Rik	RIKEN cDNA C030048B08 gene	2,03	1,02	1,69E-02

FC : Fold Change. Adujst p-Values were calculated using Benjamani-Hoschberg method.

Supplemental Table1 (B) : Down-regulated genes

Gene symbol	Gene name	Absolute (FC)	Log(FC)	adjust p-Value
Ribosome biogenesis and protein synthesis				
LOC100045542	similar to FERMRhoGEF (Arhgef) and pleckstrin domain protein 1	3,28	-1,71	3,90E-02
Rbm47	RNA binding motif protein 47 , transcript variant 1	5,43	-2,44	2,34E-02
Zc3h12d	zinc finger CCCH type containing 12D	3,02	-1,59	6,14E-02
Tgm2	transglutaminase 2, C polypeptide	2,79	-1,48	6,70E-02
Rps6ka1	ribosomal protein S6 kinase polypeptide 1	2,74	-1,45	8,48E-02
Anpep	alanyl (membrane) aminopeptidase	2,63	-1,40	9,57E-02
Acaa1a	acetyl-Coenzyme A acyltransferase 1A	2,18	-1,13	3,09E-02
Rpl21	ribosomal protein L21	2,13	-1,09	1,06E-02
Sympk	symplekin	2,22	-1,15	1,50E-02
Transcription factors and DNA-binding proteins				
Zbtb20	zinc finger and BTB domain containing 20, transcript variant 1	7,53	-2,91	8,70E-02
Dip2c	DIP2 disco-interacting protein 2 homolog C	2,63	-1,39	3,13E-02
Zfp760	zinc finger protein 760	2,29	-1,19	5,69E-02
Nfam1	Nfat activating molecule with ITAM motif 1	6,87	-2,78	3,58E-02
Nab2	Ngfi-A binding protein 2 , transcript variant 1	6,21	-2,63	5,38E-02
Rarg	retinoic acid receptor, gamma , transcript variant 1	3,59	-1,84	2,30E-02
Highly expressed in immune system				
ENSMUST00000103424	immunoglobulin heavy chain 5 (delta-like heavy chain)	25,76	-4,69	3,11E-04
Ncf1	neutrophil cytosolic factor 1	8,79	-3,14	3,01E-02
Cybb	cytochrome b-245, beta polypeptide	8,69	-3,12	5,00E-02
Csf2rb	colony stimulating factor 2 receptor, beta, low-affinity (granulocyte-macrophage)	7,59	-2,92	4,57E-02
Sema7a	sema domain, immunoglobulin domain (Ig), and GPI membrane anchor 7A	6,38	-2,67	4,34E-02
Mpeg1	macrophage expressed gene 1	5,65	-2,50	6,63E-02
H2-DMb1	histocompatibility 2, class II, locus Mb1	5,21	-2,38	2,25E-02
Lilrb3	leukocyte immunoglobulin-like receptor, subfamily B (with TM and ITIM domains), member 3	5,04	-2,33	4,79E-02
Cd300lf	CD300 antigen like family member F , transcript variant 2	4,17	-2,06	3,11E-02
LOC547349	similar to MHC class I antigen precursor	3,98	-1,99	2,06E-02
H2-T9	histocompatibility 2, T region locus 9	3,62	-1,85	2,03E-02
C1ra	complement component 1, r subcomponent A	3,60	-1,85	2,34E-02
LOC675328	similar to MHC H2-TL-T10-b	3,48	-1,80	4,86E-03
Csf2rb2	colony stimulating factor 2 receptor, beta 2, low-affinity (granulocyte-macrophage)	3,42	-1,77	1,87E-02
Irgm2	immunity-related GTPase family M member 2	3,23	-1,69	7,40E-02
Gm7229	similar to IL-1 receptor-associated kinase 2	2,82	-1,50	4,05E-02
Sh2d3c	SH2 domain containing 3C	2,57	-1,36	9,18E-02
Csf2rb	colony stimulating factor 2 receptor, beta, low-affinity (granulocyte-macrophage)	2,52	-1,33	6,88E-02
Spn	sialophorin (Spn), transcript variant 1	2,13	-1,09	9,47E-02
Intracellular signal transduction modulators and effectors				
Tbkbp1	TBK1 binding protein 1	2,52	-1,33	3,09E-02
Slc7a7	solute carrier family 7 (cationic amino acid transporter, y+ system), member 7	9,19	-3,20	5,65E-02
Slc39a4	solute carrier family 39 (zinc transporter), member 4	14,02	-3,81	6,01E-02
Hck	hemopoietic cell kinase (Hck), transcript variant 1	10,39	-3,38	8,48E-02
Ggh	gamma-glutamyl hydrolase	10,03	-3,33	1,29E-02
Sirpa	signal-regulatory protein alpha , transcript variant 1	6,75	-2,76	4,30E-02
Lxn	latexin (Lxn)	6,44	-2,69	8,67E-02
Rap1gap	Rap1 GTPase-activating protein	4,54	-2,18	3,59E-02
Rassf4	Ras association (RalGDS/AF-6) domain family member 4	3,44	-1,78	7,85E-02
Sla	src-like adaptor (Sla), transcript variant 1	3,33	-1,74	8,16E-02
H2afb3-ps	similar to vacuolar protein sorting 52	3,29	-1,72	8,41E-04
LOC100045542	similar to FERMRhoGEF (Arhgef) and pleckstrin domain protein 1	3,28	-1,71	3,90E-02
Dusp16	dual specificity phosphatase 16 , transcript variant A1	3,27	-1,71	7,85E-02
Slc37a2	solute carrier family 37 (glycerol-3-phosphate transporter), member 2 , transcript variant 1	2,97	-1,57	6,07E-03
Ctsd	cathepsin D	2,91	-1,54	9,57E-02
Arhgap9	Rho GTPase activating protein 9	2,82	-1,50	4,15E-02
Gng12	guanine nucleotide binding protein , gamma 12 , transcript variant 2	2,82	-1,49	5,57E-02
Eml2	echinoderm microtubule associated protein like 2 , transcript variant 1	2,82	-1,49	7,47E-02
Gpr18	G protein-coupled receptor 18	2,75	-1,46	9,55E-02
Ctsh	cathepsin H	2,67	-1,42	6,30E-02
Plekho2	pleckstrin homology domain containing, family O member 2	2,40	-1,27	3,21E-02
Gng12	guanine nucleotide binding protein, gamma 12 , transcript variant 2	2,40	-1,27	7,09E-02
Exoc1	exocyst complex component 1	2,10	-1,07	2,59E-02
Metabolic enzyme-related proteins				
Nrp2	neuropilin 2 , transcript variant 1	10,96	-3,45	8,20E-02
Gaa	glucosidase, alpha, acid , transcript variant 1	5,41	-2,44	6,68E-03
Nrp2	neuropilin 2 (Nrp2), transcript variant 5	4,93	-2,30	7,80E-02
B4galt6	UDP-Gal:betaGlcNAc beta 1,4-galactosyltransferase, polypeptide 6	4,73	-2,24	6,16E-02
Ppt2	palmitoyl-protein thioesterase 2	4,72	-2,24	6,06E-02
Tcn2	transcobalamin 2 , transcript variant 1	3,60	-1,85	6,96E-02
Col4a1	collagen, type IV, alpha 1	3,15	-1,66	6,22E-02
B4galt6	UDP-Gal:betaGlcNAc beta 1,4-galactosyltransferase, polypeptide 6	3,44	-1,78	7,80E-02
Igf1r	insulin-like growth factor I receptor	3,29	-1,72	1,96E-02
Anxa6	annexin A6 , transcript variant 1	3,19	-1,68	8,96E-03
Cyp4v3	cytochrome P450, family 4, subfamily v, polypeptide 3	3,91	-1,97	8,87E-02
Apobec3	apolipoprotein B , catalytic polypeptide 3, transcript variant 2	2,90	-1,54	7,51E-02
Man2a2	mannosidase 2, alpha 2	2,67	-1,42	6,34E-02
Cbr2	carbonyl reductase 2	2,33	-1,22	6,22E-02
Mthfd2l	methylenetetrahydrofolate dehydrogenase 2-like	2,32	-1,21	9,53E-02
Adcy7	adenylate cyclase 7 , transcript variant 3	2,21	-1,14	5,72E-02
Manba	mannosidase, beta A, lysosomal	2,21	-1,14	5,36E-02
Sepw1	selenoprotein W, muscle 1	2,11	-1,08	4,22E-02
Lipid metabolism				
Smpdl3a	sphingomyelin phosphodiesterase, acid-like 3A	6,97	-2,80	5,38E-02
Apoe	apolipoprotein E	13,41	-3,75	3,09E-02
St8sia6	ST8 alpha-N-acetyl-neuraminide alpha-2,8-sialyltransferase 6	9,65	-3,27	3,26E-02
Plin3	perilipin 3	5,68	-2,51	8,02E-02
Abcb1b	ATP-binding cassette, sub-family B (MDR/TAP), member 1B	5,37	-2,43	5,17E-02
Osbpl3	oxysterol binding protein-like 3 , transcript variant 2	2,76	-1,47	4,10E-02
Sgpl1	sphingosine phosphate lyase 1	2,67	-1,42	9,12E-02
Abcg1	ATP-binding cassette, sub-family G , member 1	2,63	-1,39	7,77E-02
Cell cycle				
Bcl2a1d	B-cell leukemia/lymphoma 2 related protein A1d	8,07	-3,01	5,70E-02
Bcl2a1c	B-cell leukemia/lymphoma 2 related protein A1c	5,31	-2,41	5,73E-02
Xaf1	XIAP associated factor 1	3,90	-1,96	6,84E-02
Cdk20	cyclin-dependent kinase 20	2,80	-1,49	8,15E-02
Cell adhesion-related protein (Table 2-suite)				
Nid1	nidogen 1	7,78	-2,96	5,62E-02
Rap1gap	Rap1 GTPase-activating protein	7,56	-2,92	3,49E-02
Rdx	radixin (Rdx), transcript variant 1	5,82	-2,54	9,57E-02
Anxa2	annexin A2	5,50	-2,46	3,08E-02
Kctd17	ref|Mus musculus potassium channel tetramerisation domain containing 17	4,01	-2,00	8,67E-02
Itgb1	ref|Mus musculus integrin beta 1 (fibronectin receptor beta)	3,95	-1,98	5,07E-03
Thbs3	thrombospondin 3	2,26	-1,18	8,20E-02
Blood coagulation proteins				
Hba-a1	hemoglobin alpha, adult chain 1	8,06	-3,01	3,76E-02
Hbb-b1	hemoglobin, beta adult major chain	7,92	-2,99	3,09E-02
Hba-a2	hemoglobin alpha, adult chain 2	5,92	-2,57	3,22E-02
Snca	synuclein, alpha , transcript variant 1	3,37	-1,75	5,53E-02
Mitochondrial -related protein				
Nmnat3	nicotinamide nucleotide adenylyltransferase 3	4,67	-2,22	3,09E-02
Alas2	aminolevulinic acid synthase 2, transcript variant 1	4,73	-2,24	7,85E-02
Isoc2b	isochorismatase domain containing 2b	2,49	-1,32	3,91E-02
Others				
Trim12	tripartite motif-containing 12 (Trim12), mRNA [NM_023835]	29,52	-4,88	5,41E-03
BC018473	cDNA sequence BC018473	6,96	-2,80	3,01E-02
0610042G04Rik	RIKEN cDNA 0610042G04 gene (0610042G04Rik), mRNA [XM_001472222]	5,94	-2,57	2,40E-02
ENSMUST00000100423	Putative uncharacterized proteinMCG123851	5,92	-2,56	4,57E-02
Trim12	tripartite motif-containing 12	4,34	-2,12	2,06E-02
ENSMUST00000101310	predicted gene 1070 Gene	3,50	-1,81	3,06E-02
Trim59	tripartite motif-containing 59	3,49	-1,80	3,11E-02
2010016I18Rik	RIKEN cDNA 2010016I18 gene	3,21	-1,68	8,51E-02
AA388235	expressed sequence AA388235	2,95	-1,56	3,45E-03
LOC677216	similar to vacuolar protein sorting 52	2,62	-1,39	5,64E-03
A830007P12Rik	RIKEN cDNA A830007P12 gene	2,41	-1,27	8,71E-03
9530082P21Rik	0 day neonate lung cDNA, RIKEN full-length enriched library	2,32	-1,21	6,49E-02
Gm4552	similar to keratin associated protein 5-5	2,20	-1,14	5,64E-03
9030607L17Rik	RIKEN cDNA 9030607L17 gene	2,07	-1,05	4,87E-02
Fam132a	family with sequence similarity 132, member A	2,00	-1,00	6,55E-02

FC : Fold Change. Adujst p-Values were calculated using Benjamani-Hoschberg method.

Supplemental Table 2

Top regulated network in up-regulated genes

1- Cell Cycle, Cancer, Gastrointestinal disease
Atad2, Btg1, Cort, Dnajb9, Eif2s1, Eif4a2, Gpdl1, H3f3b, Hist1h3b, Hist1h2aa, Gm12260, hist1h3f, Hist1h1e, hist2h2bb, hist1h2bg, Pkib, RPL21, Slc25a37, Tirap

2- Cellular development, Cellular growth and proliferation, Hepatic system, development and function
Abcb4, Atad2, Ehd2, Eif2s1, Gas5, Gzmm, Hist1h2ac, hist2h2be, Hsd17b7, Lgr5, Mt-nd6, Rpl17, Satb2, Tram2

3- Cell death and survival, Cell morphology, Cellular assembly and organization
Ccl25, CD69, Dup6, Eif2s1, Fosb, Hla-b, Ppp1r15a, Ptk2, pvrl2, Rbl1, Trib1

Top regulated network in down-regulated genes

1- Cellular Movement, Immune cell trafficking, Hematological System development and function
Abcb1b, anxa2, Anxa6, Bcl2a1, Col4a1, Csf2rb, Cybb, Fam132a, Hck, Igf1r, Itgb1, Lilrb3, ncf1, nid1, rap1gap, Sirpa, Snca, Spn, Tgm2, Zbtb20

2- Cellular growth and proliferation, Lymphoid tissue structure and development, Organ morphology
Adcy7, Bcl2a1c, Cbr2, Cdk20, Cybb, exoc1, Gpr18, hba1/hba2, Hck, Nrp2, Osbpl3, Plin3, ppt2, Sgpl1, Sympk, x[illegible]

3- Cancer, Cell death and survival, Gastrointestinal disease
abcb1b, abcg1, acaa1, anpep, c1r, Ctsh, Cybb, gaa, Manba, rassf4, Rbn47, rpl21, Sh2d3c, Slc7a7, thbs3

4- Development disorder, Hematological disease, Organ morphology
Abcg1, Alas2, ApoE, CD300lf, Ctsd, dusp16, Hba1/Hba2, Hbb, HLA-DMB, Ncf1, Ptprj, Rarg, Rps6Ka1, Sla

5-Hematological disease, Infectious disease, RNA damage and repair
Apobec3b, B4galt6, Cyp4v2, Gng12, Irgm2, Mthfdl2l, plekho2, rdx, Sema7a, tcn2, tgm2

6- Cell death and survival, Cell morphology, Cellular function and maintenance
ggh, Kctd17, mpeg1, nab2, slc39a4, Snca, St8sia6, Tbkbp1
